# A new species of the leafhopper genus *Maiestas* Distant from Australia (Hemiptera, Cicadellidae, Deltocephalinae, Deltocephalini)

**DOI:** 10.3897/zookeys.646.10912

**Published:** 2017-01-17

**Authors:** Yani Duan, Christopher H. Dietrich, Yalin Zhang

**Affiliations:** 1 School of Plant Protection, Anhui Agricultural University, Hefei, Anhui Province 230036, China; 2 Illinois Natural History Survey, Prairie Research Institute, University of Illinois, Champaign, IL 61820, USA; 3 Key Laboratory of Plant Protection Resources and Pest Management of the Ministry of Education, Entomological Museum, Northwest A & F University, Yangling, Shaanxi Province 712100, China

**Keywords:** Auchenorrhyncha, morphology, new species, taxonomy

## Abstract

A new leafhopper species *Maiestas irwini* **sp. n.** is described and illustrated from Australia. A checklist of the genus from the Australian region is provided together with a key to species for males.

## Introduction

The grassland leafhopper genus *Maiestas* was established by Distant (1917) with the type species *Maiestas illustris* Distant from the Seychelles. It belongs to the *Deltocephalus* group as reviewed by Webb and Viraktamath (2009), as part of a larger study of Old World Deltocephalini and re-assessment of *Maiestas* Distant. Subsequently, Zhang and Duan (2011) revised the group in China and currently the genus comprises 98 species. It differs from *Deltocephalus* Burmeister and *Recilia* Edwards by the aedeagal shaft being at most only slightly curved dorsally with its apex not notched and sometimes produced into a thin process or spine with the gonopore apical on the dorsal surface. In this paper, a new species of *Maiestas* Distant is described from Australia bringing the total for the Australian region to six species (see checklist). A checklist and a key to these species for males are provided. Images of all previously known Australian species can be seen on Fletcher’s (2016) website.

## Materials and methods

Morphological terminology follows Dietrich (2005). Digital photographs were taken with a QImaging Micropublisher 3.3 digital camera mounted on an Olympus BX41 stereo microscope and with a Nikon D1x digital SLR camera configured with lenses by Microptics, Digital Lab XLT system. Photographs were modified with Adobe Photoshop CS. Abbreviations used herein are INHS: Illinois Natural History Survey, Champaign Ill, USA; QDPI: Queensland Department of Agriculture and Fisheries, Brisbane, Australia; QM: Queensland Museum, Brisbane, Australia.

## Taxonomy

### Maiestas

Taxon classificationAnimaliaHemipteraCicadellidae

Distant

Maiestas Distant, 1917: 312. Type species: Maiestas illustris Distant, 1917, by monotypy.
Togacephalus Matsumura, 1940: 38. Type species: Deltocephalus distincta Motschulsky, 1859, by original designation.
Inazuma Ishihara, 1953: 15. Type species: Deltocephalus dorsalis Motschulsky, 1859, by original designation.
Inemadara Ishihara, 1953: 48. Type species: Deltocephalus oryzae Matsumura, 1902, by original designation.
Insulanus Linnavuori, 1960: 303. Type species: Stirellus subviridis Metcalf, 1946, by original designation.

#### Distribution.

The Old World.

#### Checklist of species of *Maiestas* Distant from the Australian region

Note: see Fletcher (2016) for full synonymy.

*Maiestas dorsalis* (Motschulsky, 1859) (Qld, NT, NSW, Oriental region)

*Maiestas irwini* **sp. n.** (Qld)

*Maiestas knighti* Webb & Viraktamath, 2009 (ACT, NSW, NT, Tas, Vic, WA, New Zealand, Papua New Guinea, Fiji, Guam)

*Maiestas lucindae* (Kirkaldy, 1907) (Qld)

*Maiestas samuelsoni* (Knight, 1976) (Norfolk Island, New Zealand (Kermadec Islands), Fiji, New Caledonia)

*Maiestas vetus* (Knight, 1975) (ACT, NSW, NT, Vic, WA, NZ)

#### Key to species of *Maiestas* Distant from the Australian region (males)

Note: male genitalia of *Maiestas lucindae* is unknown and this species is therefore omitted from the key.

**Table:** 

1	Forewing with dark zig-zag marking (Webb and Viraktamath 2009, fig. 36o)	***Maiestas dorsalis***
–	Forewing without zig-zag marking	**2**
2	Aedeagal shaft with ventral margin extending beyond gonopore by approximately 5× apical width of shaft (Webb and Viraktamath 2009, fig. 35h)	***Maiestas vetus***
–	Aedeagal shaft with ventral margin extending beyond gonopore by approximately apical width of shaft	**3**
3	Style apophysis robust (Fig. 2E)	***Maiestas irwini* sp. n.**
–	Style apophysis slim	**4**
4	Subgenital plate lateral margin slightly convex (Webb and Viraktamath 2009, fig. 39d)	***Maiestas knighti***
–	Subgenital plate lateral margin slightly concave (Webb and Viraktamath 2009, fig. 41d)	***Maiestas samuelsoni***

### Maiestas irwini sp. n.

Taxon classificationAnimaliaHemipteraCicadellidae

http://zoobank.org/439E3157-1A52-4847-9A0D-13D25C053D2C

Figs 1 , 2

#### Length.

Male: 2.6–3.0 mm.

#### Coloration and morphology.

**Figure 1.**
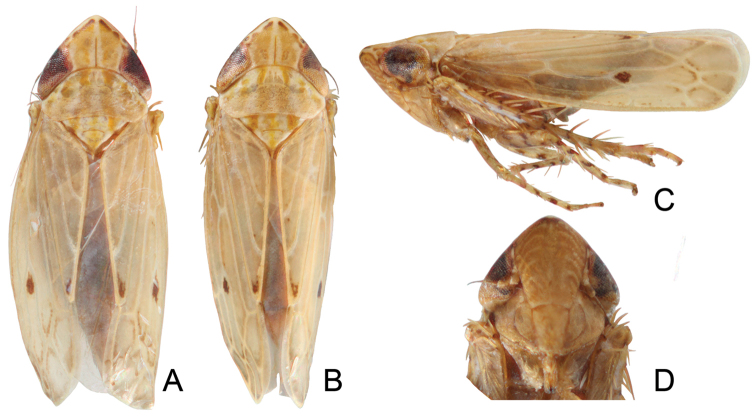
*Maiestas irwini* sp. n. **A, B** habitus, dorsal view **C** habitus, lateral view **D** face.

Ground color stramineous marked with orange and fuscous (Fig. 1A–C). Fore margin of head with fuscous marks and light fasciae extending to scutellum, coronal sulcus prominent (Fig. 1A–B). Face mostly brown, with paired white arcs corresponding to muscle scars of frontoclypeus (Fig. 1D). Pronotum with three pairs of fasciae. Scutellum with three fasciae (Fig. 1A–B). Forewing pale ochraceous, with two distinct, irregular fuscous maculae, one at the apex of the clavus and the other at the base of the central anteapical cell, veins contrastingly pale, veins of apex bordered with fuscous. Mesosternum light brown. Femora and tibiae with fuscous marks (Fig. 1C).

Head wider than pronotum, crown depressed, anterior margin distinctly angulate in dorsal view, slightly longer than distance between eyes (Fig. 1A–B). Ocellus closely adjacent to eye on anterior margin of vertex (Fig. 1A–C). Anteclypeus tapering toward the apex, not extended to ventral margin of face. Lorum semicircular, narrower than anteclypeus, well separated from lateral margin of face (Fig. 1D). Pronotum nearly as long as vertex (Fig. 1A–B). Forewing macropterous, with four apical and three anteapical cells, inner anteapical cell open basally, costal area with one cross vein (Fig. 1C).

#### Male genitalia.

**Figure 2.**
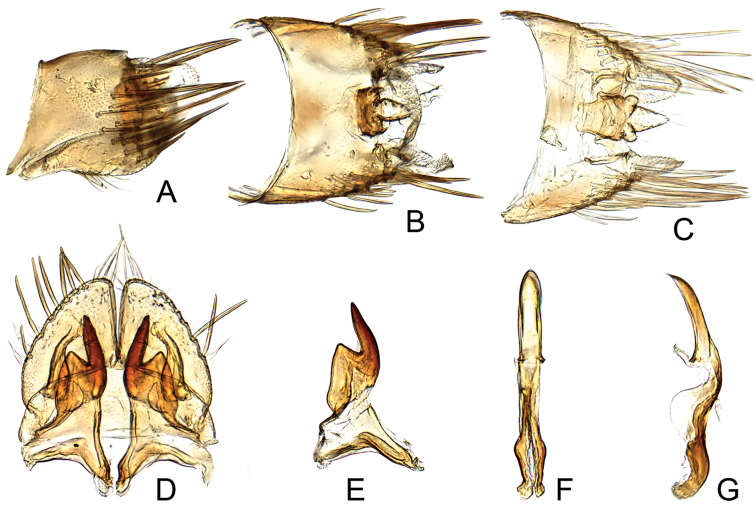
*Maiestas irwini* sp. n. **A** male pygofer lobe, lateral view **B, C** male pygofer and segments X–XI, dorsal view **D** valve, subgenital plates and styles, ventral view **E** style, dorsal view **F, G** connective and aedeagus, dorsal and lateral view, respectively.

Pygofer lobe with numerous apical macrosetae, longer than its height, hind margin rounded (Fig. 2A–C). Subgenital plate subtriangular, lateral margin convex, length nearly as long as width. Valve rectangular (Fig. 2D). Style preapical lobe angulated, apophysis digitate, slightly laterally curved (Fig. 2E). Connective slightly longer than aedeagus. Aedeagal shaft short, stout, more or less of uniform width, curved dorsally with ventral margin produced into small spine beyond gonopore (Fig. 2F–G).

#### Material examined.

**Holotype**: 1 male, 4km up Black Mountain Road, via Kuranda, 14.ix.–12.x.1982, malaise trap (QM, T234944, ex QDPI). **Paratypes**: 1 male, same data as holotype (QDPI); 2 males, same data as previous but 14.ix–12.x.1982, G. Simpson (QDPI); 1 male, 1 female, same data as holotype but 12–26.x.1982 (QDPI); 3 males, 3 females, Moggill State Forest, 26 km W Brisbane, Queensland, 17.x.1983, M. E. Irwin, malaise trap in gully in eucalyptus (INHS); 1 male, Mount Baldy Rd via Atherton, N Queensland, vi.1981, J. D. Brown, malaise trap (QDPI); 1 male, Tully Falls Rd, 10.iii.1956, J. L. Gressitt, light trap (BPB).

#### Remarks.

The male genitalia of this species are similar to those of *Maiestas scriptus* (Distant), from India (Webb & Viraktamath, 2009, Fig. 33) with a short and broad subgenital plate with lateral margin well rounded (Fig. 2D), style apophysis relatively long and straight (Fig. 2E), and aedeagal shaft short (Fig. 2F–G), but *Maiestas irwini* differs in color pattern, the more strongly produced head (Fig. 1A–B), and less acute aedeagal apex in dorsal view (Fig. 2F). The new species differs from other Australian species (see Fletcher, 2016) in coloration and genital morphology.

#### Etymology.

This species is named for M. E. Irwin who collected much of the type series.

## Supplementary Material

XML Treatment for Maiestas

XML Treatment for Maiestas irwini
